# Effects of Xingnaojing Injection on Adenosinergic Transmission and Orexin Signaling in Lateral Hypothalamus of Ethanol-Induced Coma Rats

**DOI:** 10.1155/2019/2389485

**Published:** 2019-06-27

**Authors:** Xiao-Tong Chen, Xiao-Ge Wang, Li-Yuan Xie, Jia-Wen Huang, Wei Zhao, Qi Wang, Li-Mei Yao, Wei-Rong Li

**Affiliations:** ^1^Institute of Clinical Pharmacology, Guangzhou University of Chinese Medicine, 12 Jichang Road, Baiyun District, Guangzhou 510405, China; ^2^School of Traditional Chinese Medicine Healthcare, Guangdong Food and Drug Vocational College, 321 Longdong North Road, Tianhe District, Guangzhou 510520, China

## Abstract

Acute alcohol exposure induces unconscious condition such as coma whose main physical manifestation is the loss of righting reflex (LORR). Xingnaojing Injection (XNJI), which came from Chinese classic formula* An Gong Niu Huang* Pill, is widely used for consciousness disorders in China, such as coma. Although XNJI efficiently shortened the duration of LORR induced by acute ethanol, it remains unknown how XNJI acts on ethanol-induced coma (EIC). We performed experiments to examine the effects of XNJI on orexin and adenosine (AD) signaling in the lateral hypothalamic area (LHA) in EIC rats. Results showed that XNJI reduced the duration of LORR, which implied that XNJI promotes recovery form coma. Microdialysis data indicated that acute ethanol significantly increased AD release in the LHA but had no effect on orexin A levels. The qPCR results displayed a significant reduction in the Orexin-1 receptors (OX_1_R) expression with a concomitant increase in the A_1_ receptor (A_1_R) and equilibrative nucleoside transporter type 1 (ENT1) expression in EIC rats. In contrast, XNJI reduced the extracellular AD levels but orexin A levels remained unaffected. XNJI also counteracted the downregulation of the OX_1_R expression and upregulation of A_1_R and ENT1 expression caused by EIC. As for ADK expression, XNJI but not ethanol, displayed an upregulation in the LHA in EIC rats. Based on these results, we suggest that XNJI promotes arousal by inhibiting adenosine neurotransmission via reducing AD level and the expression of A_1_R and ENT1.

## 1. Introduction

Acute alcohol binge drinking leading to ethanol-induced coma (EIC) has become extremely prevalent [1], the main symptom which is the loss of righting reflex (LORR). The economic burden caused by alcohol is almost to be 1-3% of total health costs in the global [2]. Extensive studies suggest that EIC has negative effects on many structures and their functions. EIC impairs cognitive control and hematopoietic tissues and increases the risk of infectious diseases particularly pneumonia [3–6]. In addition, alcohol causes many health problems, including traumatic brain injury, liver disease and cancer [7–9]. Since alcohol is available to anyone, both acute alcohol and chronic alcohol are paired with negative consequences in individuals, families, and society [10, 11].

The lateral hypothalamic area (LHA), which is rich in orexin neurons, plays a central role in the regulation of arousal. To some degree, the wakefulness promotion of the LHA is attributed to orexin neurons. Orexin A and orexin B, derived from the same precursor, are synthesized in these neurons and bind to two G-protein coupled receptors, orexin-1 and orexin-2 [12]. Orexin-1 receptors (OX_1_R) selectively bind to orexin A, whereas orexin-2 receptors (OX_2_R) show the equal affinity to both orexin A and orexin B [13]. Orexin system is involved to physiological functions including sleep-wakefulness, energy homeostasis, and pathological states, such as coma and drug abuse [14, 15]. The loss of orexin neurons or OX_1_R blockade results in a reduced wakefulness and prolonged total sleep time [16, 17]. In contrast, activation of orexinergic transmission, such as orexin A administration, exerts wakefulness promotion [18]. Although most studies have identified that the orexin signaling promotes wakefulness, how the orexin transmission acts on EIC still remains poorly understood.

Adenosine (AD), a sleep-promoting neuromodulator, has a role in mediating many neuronal and behavioral effects of ethanol [19–21]. Considerable evidence suggests that acute ethanol increases the extracellular AD level acted on the A_1_ receptor (A_1_R) and A_2A_ receptor (A_2A_R) to inhibit the wakefulness-maintaining neurons [22–26]. Among adenosine receptors contributing to sleep induction, A_1_R and A_2A_R have been widely observed in many studies [22, 26–28]. Administration of the selective A_1_R antagonist DPX or DPCPX reduces the nonrapid eye movement (NREM) sleep [21, 25], and administration of the selective A_2A_R agonist CGS21680 increases the NREM sleep [29].

Ethanol is well-known to increase the extracellular adenosine levels [21]. One reason why acute ethanol increases the extracellular AD is to inhibit AD uptake via equilibrative nucleoside transporter type 1 (ENT1) which is a bidirectional transporter for nucleosides including AD [30]. Ethanol dependent rats display a significant reduction in both A_1_R and ENT1 expression in the basal forebrain during withdrawal [31]. Furthermore, Choi and colleagues suggest that the increase in voluntary ethanol intake in ENT1-null mice may be due to the profound decrease in A_1_R signaling but not a defect in A_1_R function [32]. In addition, inhibition of adenosine kinase (ADK) reduces the AD metabolisms, which may also induce increased extracellular AD levels.

Xingnaojing injection (XNJI), extracted from a classic Chinese emergency prescription called* An Gong Niu Huang *Pill, is one of the most widely used traditional Chinese Medicine Patent Prescription in emergency room in China. The function of XNJI is related to clean heat-toxic, promote blood flow and ameliorate brain function [33]. XNJI has good therapeutic effects on the consciousness diseases including stroke and all kinds of coma in both clinical trials and experimental studies because of its brain protection [34–36]. According to meta-analysis, XNJI has a significant benefit on recovery compared to conventional drugs treatment in unconscious patients by improving hemorrheology and neurological deficit, reducing serum TNF-*α* level [35, 37, 38]. EIC induces a sudden unconscious condition that threatens one or more organic systems. Although XNJI is the most commonly used emergency aid for hangover in China, little is known about the neural mechanism mediating the effects of XNJI on EIC.

We have previously shown that acute ethanol intake causes a decrease in extracellular glutamate and increase in extracellular *γ*-aminobutyric acid (GABA) in the LHA which are reversed by XNJI treatment [39]. In addition to glutamate/GABA signaling, we hypothesized that XNJI may play an important role in EIC by altering adenosine neurotransmission and orexin signaling. Therefore, we focused our attention on understanding the role of adenosine and orexin signaling on EIC following XNJI administration.

## 2. Materials and Methods

### 2.1. Chemicals and Drugs

The absolute ethyl alcohol was purchased from Guangzhou chemical reagent factory (Guangzhou, China). A 34% (v/v, in water) solution was made fresh on the day of the experiment and administered at a dose of 3.76 g/kg. Artificial cerebrospinal fluid (ACSF = NaCl 145 mM, KCl 2.7 mM, MgCl_2_ 1.0 mM, and CaCl_2_ 1.2 mM; pH=7.4) was prepared fresh on the day of the experiment. All salts used for ASCF preparation were purchased from Guangzhou chemical reagent factory (Guangzhou, China). Adenosine standards was purchased from Purechem-Standard Co., Ltd. (Chengdu, China). XNJI was purchased from Jiminkexin Pharmaceutical Company (batch number: 161216; Wuxi, China) with the China Food and Drug Administration number Z32020562. XNJI is approved by China Food and Drug Administration [34] and its components are as follows: 1 mL XNJI contains 7.5 mg Moschus (*Moschus berezovskii* Flerov), 1 mg borneolum (*Blumea balsamifera* (L.) DC.), 30 mg Curcumae radix (*Curcuma aromatica *Salisb), and 30 mg Gardeniae fructus (*Gardenia jasminoides *J. Ellis), which suggests that the concentration of XNJI is 68.5 mg/mL. To guarantee the quality and stability of the XNJI, we assayed the volatile components in* Moschus *and borneol by gas chromatography (GC) and nonvolatile components in* Curcuma aromatic* Salisb and* Gardenia jasminoides* J. Ellis by high performance liquid chromatography (HPLC). The concentrations of the four volatile components were 0.087 mg/mL muscone, 1.11 mg/mL borneol, 0.054 mg/mL isoborneol, and 0.078 mg/mL camphor. The concentrations of the four nonvolatile components were 2.83 *μ*g/mL jasminoidin, 0.36 *μ*g/mL curcumin, 0.14 *μ*g/mL demethoxycurcumin, and 0.50 *μ*g/mL bisdemethoxycurcumin. For the detailed protocol conditions see our previous experiment [39]. The voucher specimens were deposited at Institute of Clinical Pharmacology, Guangzhou University of Chinese Medicine (Guangzhou, China).

### 2.2. Animals and Grouping

Adult male Sprague-Dawley rats (200 g-300 g, Guangdong Medical Experimental Animal Center, China) were housed in an animal facility with ambient temperature and humidity and* ad libitum* access to food and water. Rats were randomly assigned to five groups (n=6 per group): control group, ethanol-induced coma group (EIC), low dose of XNJI group (XNJI-L), middle dose of XNJI group (XNJI-M), and high dose of XNJI group (XNJI-H). All experimental protocols were conducted according to the National Research Council Guide for the Care and Use of Laboratory Animals and approved by the Animal Experimentation Committee at the Guangzhou University of Chinese Medicine.

### 2.3. Surgery

Anesthetized with chloral hydrate (10%; 0.35 g/kg; i.p.), the rats were placed on the stereotaxic apparatus (Ruiwode, Shenzhen, China). A guide cannula (CMA, Stockholm, Sweden) was implanted unilaterally at 90° angle above the LHA (target coordinates were: AP= -3.3 mm, ML= -1.5 mm, DV= -8.5 mm, including probe membrane length [40]). After the surgery, penicillin sodium was administrated subcutaneously, used as an antibiotic to prevent infection.

### 2.4. Ethanol Consumption, XNJI Administration and Microdialysis Sampling

After 2 days of postoperative recovery, a microdialysis probe (CMA/12, 2 mm membrane length; CMA Microdialysis, Stockholm, Sweden) was inserted into the LHA through the guide cannula. Before ethanol and XNJI administration, ACSF was perfused at a flow rate = 2 *μ*L/min with a CMA 402 microdialysis pump (CMA, Stockholm, Sweden). The delay between the time adenosine and orexin A diffused into the probe and when the dialysate reached the tubing outlet is 9 min. 2 × 45min pretreatment baseline samples (90 *μ*L/ sample) were collected after one-hour perfusion for equilibrium. Then, the control rats were given saline i.p. while others were treated with ethanol [34% (v/v, in water); 3.76 g/kg, i.p.] 20 minutes before XNJI microinjection. Subsequently, the saline and three doses of XNJI were given by unilaterally intracerebroventricular (i.c.v.) injection via the stereotaxic apparatus, respectively. Briefly, control group and EIC group were treated with saline (10*μ*L/kg), XNJI-L group was treated with 0.34mg/kg XNJI, XNJI-M group was given 0.68mg/kg XNJI, and XNJI-H group was given 1.36mg/kg XNJI according to the body weight. Subsequently, ACSF was perfused and 6 × 45 min posttreatment samples were collected. The flow rate was maintained at 2 *μ*L/min during the whole experiment. The dialysate samples were stored in ice until analyzed. On completion, animals were euthanized and hypothalamus was removed and stored in −80°C for the further analysis of gene expression.

### 2.5. Loss of Righting Reflex Test

Rats were treated with ethanol [34% (v/v, in water); 3.76 g/kg] by intraperitoneal administration (i.p.), which results in the LORR in animals. LORR means to fail to correct its posture while lying on its back. Duration of LORR was the time interval that measured from the appearance of LORR to recovery of righting reflex after acute ethanol exposure.

### 2.6. Measurement of Extracellular Adenosine and Orexin A

The microdialysis samples were analyzed by HPLC coupled with an ultraviolet (UV) detector [24, 41]. In brief, 20 *μ*L samples were injected into the HPLC. Adenosine was separated out with a Gemini 5 *μ* C18 column (250 × 4.6 mm, Phenomenex, CA, USA) and detected by UV detector (Agilent 1260 infinity, Agilent Technologies Inc.; California, USA) at 260 nm wavelength. The mobile phase contained 8 mm NaH_2_PO_4_ and 20% methanol (flow rate = 1 mL/min) revised by others [42]. The chromatogram data was acquired and analyzed by Agilent OpenLab system (Agilent Technologies Inc.; California, USA). Comparing its retention time and area under the peak to the AD standards, adenosine peak in the microdialysis samples was identified and quantified.

Another 50 *μ*L samples were used to measure orexin A levels with an orexin A ELISA Kit (Cusabio Biotech Co., LTD, Wuhan, China) following manufacturer' s instructions.

### 2.7. Effects of XNJI on OX_1_ R, A_1_R, ENT1, and ADK Gene Expression in Acute Ethanol Exposed Rats

Total RNA was extracted from tissue samples by Trizol reagent (Takara, Dalian, China). The protocol in detail was described in previous study [39]. In brief, RNA was extracted by Trizol reagent and purified by chloroform and isopropanol followed by washing with 75% ethanol. Subsequently, RNA was dissolved with RNase-free water. The RNA purity and concentration were analyzed with Nano UV-3000 (Thermo Fisher Scientific, MA, USA) and the concentration was used to calculate the amount of RNA needed.

Total RNA was reverse transcribed to cDNA with Bestar™ qPCR RT Kit (DBI® Bioscience, Shanghai, China) as instructions. The cDNA was used to process for real-time PCR. All cDNA samples were run in triplicate and the Bestar™ qPCR MasterMix (DBI® Bioscience, Shanghai, China) was used as instructions. OX_1_R, A_1_R, ENT1, and ADK were the target genes and *β*-actin, the housekeeping gene, was used as an internal control. All primers were designed by Doclab Biotechnology Co., Ltd. (Guangzhou, China). For each sample, a 20 *μ*L reaction mixture containing 10 *μ*L of Bestar ® SybrGreen qPCR mastermix (DBI® Bioscience), 0.5 *μ*L of forward primer (10 *μ*M), 0.5 *μ*L of reverse primer (10 *μ*M) (primer sequences are described in Table 1), 1 *μ*L of cDNA, and 8 *μ*L RNase-free water were prepared and then performed to amplification using Applied Biosystems StepOnePlus™ Real-Time PCR System (Foster city, CA, USA).

The relative fold change in mRNA expressions among control, EIC, and three experimental group animals was calculated by the 2^−∆∆Ct^ method [14, 43].

### 2.8. Statistical Analysis

One-way analysis of variance (ANOVA; SPSS, Chicago, USA) followed by the LSD* post hoc* test was performed to examine the effect of local XNJI administration on extracellular orexin A and AD release and gene expression in the LHA. All data were shown as mean ± standard error of the mean (SEM).* P*< 0.05 is the level of significance.

## 3. Results

### 3.1. Loss of Righting Reflex

**Figure 1:**
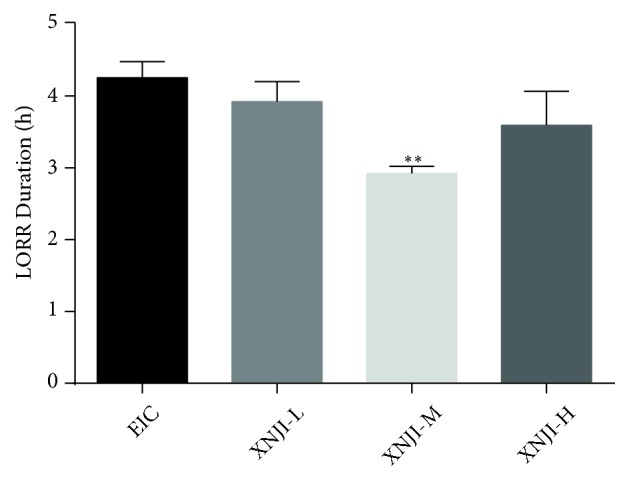
*Effect of XNJI on LORR duration following acute ethanol exposure*. Duration of LORR was significantly shortened after 0.68 mg/kg XNJI treatment (*P* <0.05, LSD* post hoc* test). See text for details. *∗ P* <0.05 and *∗∗ P* <0.01, versus the EIC.

One-way ANOVA revealed a difference of duration of LORR among groups (*F*= 3.564, df (total)= 23,* P *<0.05; Figure 1). Further Fisher's LSD analysis indicated that posttreatment with XNJI-M significantly shortened the duration of LORR (Mean±SEM= 2.92±0.24,* P* <0.05; LSD* post hoc* test) as compared to EIC group (Mean±SEM= 4.26±0.56), whereas XNJI-L (Mean±SEM=3.92±0.68) and XNJI-H (Mean± SEM= 3.59±1.16) did not display any significant decrease in LORR duration (*P* >0.05; LSD* post hoc* test).

### 3.2. Effect on Levels of Orexin A in LHA

**Figure 2:**
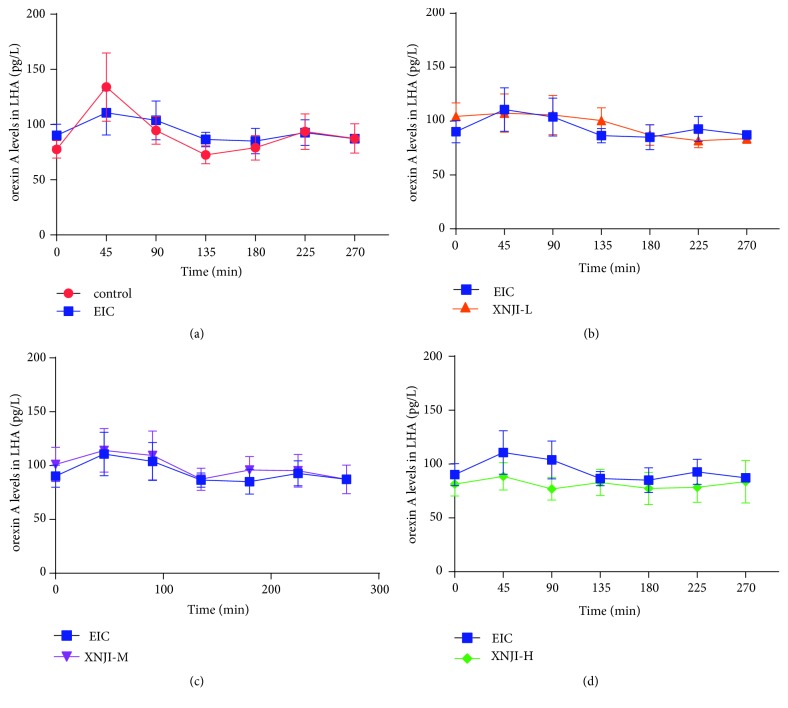
*Effect of XNJI on orexin A level in the LHA*. (a) As compared to control animals, the LHA orexin A level in ethanol-treated rats did not display a significant increase or decrease (*P* >0.05; LSD* post hoc* test;* n*=6). (b-d) As compared to EIC, orexin A level after XNJI posttreatment also remained unchanged (*P* >0.05; LSD* post hoc* test). Furthermore, orexin A concentration in each group was at a stable level during the experiment. See text for details.

The baseline orexin A levels (Mean±SEM;* n*=6) in each group were as follows: control group = 77.7±7.9 ng/L; EIC group = 90.2±10.2 ng/L; XNJI-L = 104.5±12.4 ng/L; XNJI-M = 101.1±15.9 ng/L; XNJI-H = 81.5±11.1 ng/L. One-way ANOVA depicted that a nonsignificant effect was observed in extracellular orexin A levels among five groups (Figure 2). As compared to control group, the orexin A levels in the LHA in EIC group remained unaffected (*P* >0.05; LSD* post hoc* test; Figure 2(a)). Post-XNJI treatment did not display any change in orexin A levels during the whole experiment (*P* >0.05; LSD* post hoc* test). These results demonstrate that orexin A levels do not decrease with acute ethanol intake and that the increased duration of LORR in these rats occurs independently of orexin A levels in the LHA.

### 3.3. Effect on Levels of AD in LHA

**Figure 3:**
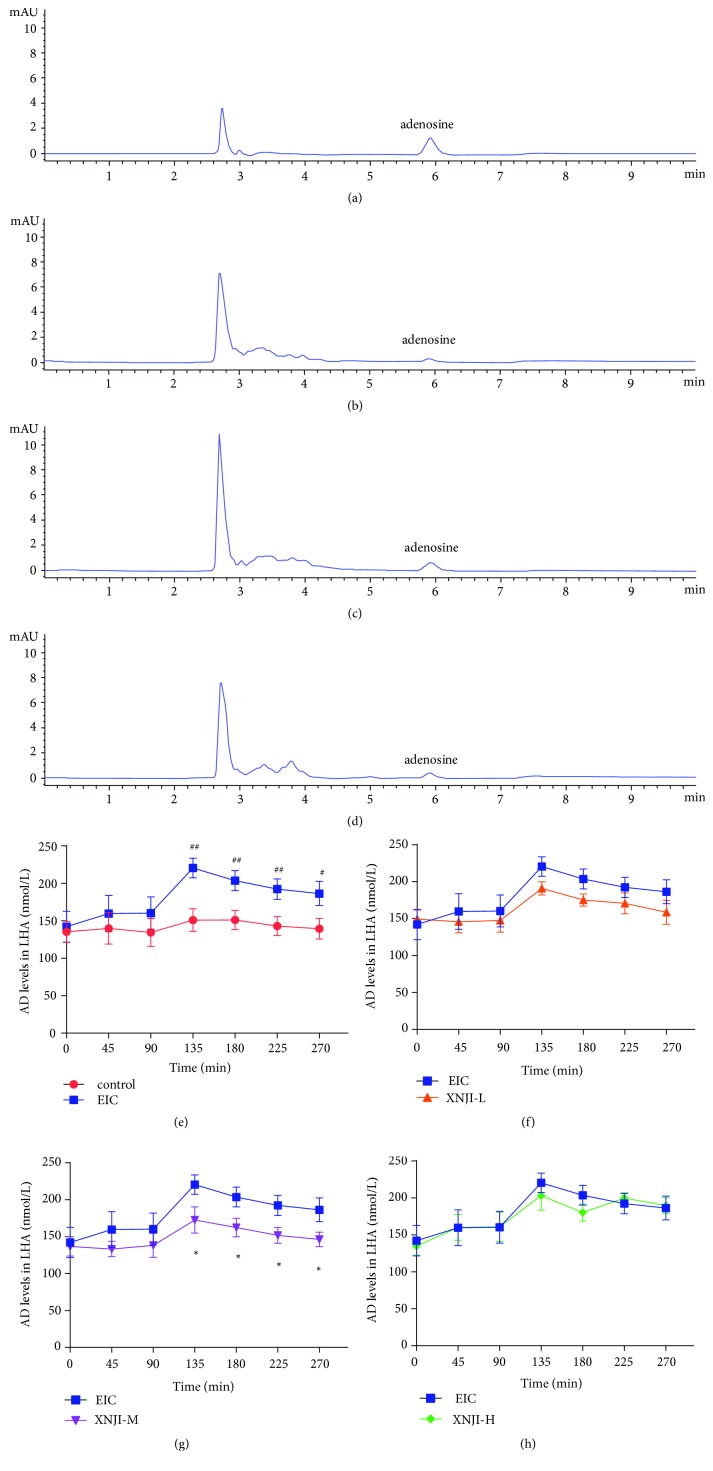
*Effect of XNJI on AD release in the LHA*. (a-d) represents HPLC chromatograms of standard and AD at 135 min after administration in control, EIC, and XNJI-M, respectively. Comparing the retention time and area under the peak to the AD standards, AD peak in the dialysates was identified and quantified. As compared to the area under the peak of control, that of EIC was increased. As compared to the EIC, the area under the peak of XNJI-M was decreased. (e-h) depict the hourly profile of AD level during the experiment. As compared to control animals, ethanol-treated rats displayed a significant increase in the LHA AD level. (*P* <0.05; LSD* post hoc* test;* n*=6). As compared to EIC, XNJI-M significantly decreased AD release in the LHA (*P* <0.05, LSD* post hoc* test). See text for details. ^#^* P* <0.05 and ^##^* P* <0.01, versus the control group; *∗ P* <0.05 and *∗∗ P* <0.01, versus the EIC.

Comparing the retention time and area under the peak to the AD standards, AD in the dialysates was identified and quantified (Figures 3(a)–3(d)). As compared to the area under the peak of control, that of EIC was increased. As compared to the EIC, the area under the peak of XNJI-M was decreased, which suggests that ethanol increased the extracellular AD level and XNJI decreased the AD levels. The baseline AD levels (Mean±SEM;* n*=6) in each group were as follow: control group = 135.3±14.1 nmol/L; EIC group = 142.1±20.5 nmol/L; XNJI-L = 149.5±12.0 nmol/L; XNJI-M = 136.9±13.1 nmol/L; XNJI-H = 134.8±12.2 nmol/L. There was no significant difference in baseline extracellular AD levels among groups. The control group did not display any change in AD levels during the whole experiment, whereas EIC group showed a significant increase in AD levels during the first 135 minutes. Although the AD levels during the postethanol perfusion were higher (*P* <0.05; LSD* post hoc* test) than the baseline AD levels, they were in a steady decline (Figure 3(e)). One-way ANOVA indicated XNJI-M significantly decreased the levels of extracellular AD during the last 135 minutes (*P* <0.05; LSD* post hoc* test; Figure 3(g)) as compared to the EIC group. Although there was a decrease in AD levels in XNJI-L group, it did not reach significance (*P* >0.05; LSD* post hoc* test; Figure 3(f)). The AD levels in XNJI-H group and EIC group were comparable during the experiment (*P* >0.05; LSD* post hoc* test; Figure 3(h)).

### 3.4. Effect on Expression of OX_1_R, A_1_R, ENT1, and ADK in LHA

**Figure 4:**
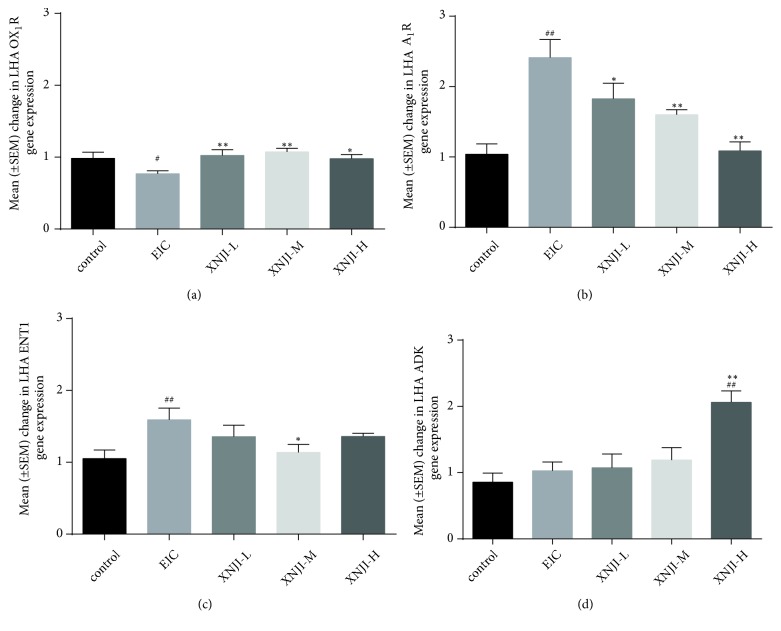
*Effect of XNJI on the LHA OX*_*1*_ *R, A*_*1*_ *R, ENT1, and ADK gene expression following acute ethanol intake*. (a-d) display the changes of OX_1_R, A_1_R, and ENT1 gene expression among five groups respectively. Acute ethanol exposure produced a significant decrease (*P* <0.05; LSD* post hoc* test; n=6) in OX_1_R expression coupled with an increase (*P* <0.01; LSD* post hoc* test) in A_1_R and ENT1 expression. However, unilateral injection of XNJI into the lateral ventricle blocked ethanol-induced downregulation of OX_1_R expression and upregulation of A_1_R and ENT1 expression. Furthermore, XNJI-H significantly upregulated ADK expression (*P* <0.01; LSD* post hoc* test). See text for details. ^#^* P* <0.05 and ^##^* P* <0.01, versus the control group; *∗ P* <0.05 and *∗∗ P* <0.01, versus the EIC.

During the experiment, there was a significant reduction in OX_1_R expression (*P* <0.05, LSD* post hoc* test) and increase in A_1_R expression (*P* <0.01, LSD* post hoc* test) in the LHA of EIC group as compared to control group. As compared to EIC group, all doses of XNJI induced a significant increase in OX_1_R expression (*P* <0.05; LSD* post hoc* test; Figure 4(a)) and dose-dependently decreased A_1_R expression (*P* <0.05; LSD* post hoc* test; Figure 4(b)). In addition, ethanol also upregulated ENT1 expression in the LHA in EIC group whereas ENT1 expression was downregulated in XNJI-M group (*P* <0.05; LSD* post hoc* test; Figure 4(c)). Interestingly, the upregulation of ADK expression was not induced by ethanol but XNJI-H (*P* <0.01; LSD* post hoc* test; Figure 4(d)).

## 4. Discussions

This is the first study implicating an interaction of XNJI and EIC via AD and orexin signaling. The results of our study demonstrate that (1) XNJI reduced the duration of LORR; (2) acute ethanol and XNJI do not affect the extracellular orexin A levels in the LHA; (3) acute ethanol intake increases the extracellular AD release which can be rescued by XNJI; (4) XNJI offsets the downregulation of OX_1_R and ADK gene expression and upregulation of A_1_R and ENT1 gene expression in the LHA caused by EIC. Based on these results, we believe that XNJI may perform wake-promotion effects mainly by reducing AD signaling.

We performed experiments at light onset. On one hand, humans usually drink at night and rats are active at night but sleep at day, so we performed experiments during daytime to imitate human behaviors. On the other hand, extracellular AD levels are highest at light onset. In contrast, AD levels are the lowest at dark onset, which makes it difficult to monitor XNJI induced reduction of extracellular AD in the LHA. Furthermore, extracellular orexin A level may be the highest at dark onset and may not increase further after XNJI administration following ethanol intake. In order to amplify the efficacy of XNJI, we conducted our experiments during the light period when the rats are minimally active. In our experiment, XNJI was administrated by i.c.v. because it allows for delivering drugs with precise control in specific brain.

Our results showed that XNJI shortened the duration of LORR, which means that XNJI promotes wakefulness. XNJI did not display a significant increase in extracellular orexin A, whereas OX_1_R gene expression in the LHA was significantly increased after XNJI administration in ethanol-treated rats, suggesting that both XNJI and ethanol have no effect on extracellular orexin A but alter the expression of OX_1_R in the LHA, which may act on the recovery of EIC. Jia et al. found that both orexin A and orexin B decreased the duration of LORR caused by ethanol [18]. Endogenous orexin A level in LORR condition is very low that may be difficult to be detected [44–47]. OX_1_R antagonism has a therapeutic effect on stress and hyperarousal states, which means that OX_1_R plays a role in arousal [48]. Orexin A levels are low in daytime in rats, so orexin A in EIC rats may be even lower that XNJI may not cause any changes to orexin A levels in the LHA. Even though all doses of XNJI increased OX_1_R gene expression, the small size effect would not activate orexin signaling without an increased orexin A levels.

Our experiments were designed to examine whether XNJI conducts arousal effects following acute ethanol exposure. Strong and compelling evidence suggests that ethanol acts directly in the brain to increase extracellular AD [31, 49], which is consistent with our results. Neurotransmitters that mediate the sleepiness and wakefulness are regulated by two interrelated regulatory processes: circadian and homeostatic [50, 51]. Jia et al. found that the EEG delta power was almost immediately increased after EIC [18]. EEG delta power, an electrophysiological indicator of slow wave sleep [52], directly represents the brain activity and is more easily monitored for short-term changes. AD, a biochemical indicator of sleep, is regulated by multiple factors and its concentration is the final result of the neurotransmission regulation. So its concentration may not change in a short period of time. In addition, XNJI may counteract the increased AD transmission caused by EIC at first 135 min, but the effect of XNJI is greater than the effect of EIC in the last 135 min.

As is known, AD, a breakdown product of ATP metabolism, corresponds to increased sleep pressure. Since acute ethanol increases extracellular AD levels whereas the AD levels in control group remain unchanged in the whole experiment, it is unlikely that the increase of AD levels is the result of sleep pressure. Furthermore, both the influx and efflux contribute to the homeostatic control of extracellular AD, which indicates that the accumulation of extracellular AD is the result of increased ATP metabolism or decreased AD uptake. Nagy and colleagues suggest that ethanol increases extracellular AD by inhibiting AD uptake via the nucleoside transporter [30]. AD can permeate biological membranes via selected AD transporter proteins. ENT1 is relatively well-developed in pharmacology as compared to other equilibrative transporter proteins and has often been identified as a major player in purinergic signaling. ENT1 mediates both AD influx and efflux [53, 54]. Our results suggest that acute ethanol upregulates ENT1 expression but does not affect ADK expression which contributes to AD metabolism, while XNJI-M downregulates ENT1 expression and XNJI-H upregulates ADK expression after EIC happened. These results imply that acute ethanol may increase extracellular AD by promoting efflux and XNJI may eliminate extracellular AD via promoting influx and intercellular metabolism.

It is reported that AD, via A_1_R, interacts with orexin signaling to promote sleep [55, 56]. In addition, A_2A_R is also responsible for sleep induction [29, 57]. Because A_2A_Rs are rich in the striatum and have low expression in the LHA, so we did not measure the A_2A_R expression in our experiments. Our results showed that XNJI attenuates the EIC via decreasing extracellular AD levels and downregulating the A_1_R expression, suggesting that XNJI acts on arousal promotion by inhibiting A_1_R activation in the LHA. These findings clearly demonstrate the importance of adenosinergic mechanisms in altering EIC.

ATP plays a vital role in sleep-wake regulation and may dedicate to extracellular AD levels [58–60]. Does ethanol mediate ATP changes in the brain? Does XNJI affect the brain energy levels to mediate extracellular AD? Can we get the same results in other sleep-wake centers? These are interesting, yet unanswered, questions that we will address in our further studies. Our results did not show a significant effect of XNJI on orexin signaling, but it does not mean that XNJI have no effects on regulation of orexin system. We need to do more experiments to draw a conclusion on whether XNJI acts on orexin signaling.

## 5. Conclusions

In our studies, orexin A remained unchanged in the whole experiment but the expression of OX_1_R had significant changes among groups. In addition, XNJI acts on adenosinergic mechanisms to promote arousal via altering extracellular AD levels and the expression of A_1_R and ENT1. Based on these results, we may draw a conclusion that XNJI promotes recovery from EIC by inhibiting adenosine neurotransmission via reducing AD level and the expression of A_1_R and ENT1.

## Figures and Tables

**Table 1:** Genes and their sequence of the primers.

Oligo Name	Sequence(5′-3′)
OX_1_R-F	GATGCTGATGGTGGTTCTG
OX_1_R-R	CAGGAGAATGCAGCCTTGAA
A_1_R-F	AACATTGGGCCACAGACCTA
A_1_R-R	GACTCGGAGGTATCGATCCA
ENT1-F	CGGAGCCTCACAGCTATTTG
ENT1-R	GCCATGAAGGTGATGAACCA
ADK-F	GCATTTGTAGGAGGGTTTCT
ADK-R	GTACAGCCAGTTCGCCTAAT
*β*-actin-F	AGGGAAATCGTGCGTGACAT
*β*-actin-R	GAACCGCTCATTGCCGATAG

## Data Availability

All original data in this manuscript are available from the author.

## Abbreviations

ACSF: Artificial cerebrospinal fluid
AD: Adenosine
ADK: Adenosine kinase
A_1_R: A_1_ receptor
ENT1: Equilibrative nucleoside transporter type 1
GABA: *γ*-aminobutyric acid
HPLC: High performance liquid chromatography
i.c.v.: Intracerebroventricular
LHA: Hypothalamic area
OX_1_R: Orexin-1 receptors
qPCR: Quantitative real-time PCR
XNJI: Xingnaojing injection.

## Additional Points

*List of Compounds*. Muscone, borneol, isoborneol, camphor, jasminoidin, curcumin, demethoxycurcumin, and bisdemethoxycurcumin
